# Developmental trajectories of executive functions in 22q11.2 deletion syndrome

**DOI:** 10.1186/s11689-016-9141-1

**Published:** 2016-03-25

**Authors:** Johanna Maeder, Maude Schneider, Mathilde Bostelmann, Martin Debbané, Bronwyn Glaser, Sarah Menghetti, Marie Schaer, Stephan Eliez

**Affiliations:** Office Médico-Pédagogique Research Unit, Department of Psychiatry, University of Geneva School of Medicine, Geneva, Switzerland; Center for Contextual Psychiatry, Department of Neuroscience, KU Leuven, Leuven, Belgium; Adolescence Clinical Psychology Research Unit, Faculty of Psychology and Educational Sciences, University of Geneva, Geneva, Switzerland; Stanford Cognitive and Systems Neuroscience Laboratory, Stanford University School of Medicine, California, USA; Research Department of Clinical, Educational and Health Psychology, University College London, London, UK; Department of Genetic Medicine and Development, University of Geneva, Geneva, Switzerland

**Keywords:** 22q11.2 deletion syndrome, Executive functions, Development, Adaptive functioning

## Abstract

**Background:**

22q11.2 deletion syndrome (22q11.2DS) is a genetic disorder associated with a specific cognitive profile. Higher-order cognitive skills like executive functions (EF) are reported as a relative weakness in this population. The present study aimed to delineate the developmental trajectories of multiple EF domains in a longitudinal sample using a broader age range than previous studies. Given the high incidence of psychotic symptoms in 22q11.2DS, we also compared the development of EF in participants with/without comorbid psychotic symptoms. Given the importance of EF in daily life, the third aim of the study was to characterize the link between EF and adaptive functioning.

**Methods:**

The sample consisted of 95 individuals with 22q11.2DS and 100 typically developing controls aged 6–26 years. A large proportion of the sample (55.38 %) had multiple time points available. Between-group differences in the developmental trajectories of three subdomains of EF (verbal fluency, working memory, and inhibition) were examined using mixed models regression analyses. Analyses were repeated comparing only the 22q11.2DS group based on the presence/absence of psychotic symptoms to investigate the influence of executive dysfunction on the emergence of psychotic symptoms. Hierarchical stepwise regression analyses were also conducted to investigate the predictive value of EF on adaptive functioning.

**Results:**

We observed lower performance on EF domains, as well as atypical development of working memory and verbal fluency. Participants who presented with negative symptoms exhibited different developmental trajectories of inhibition and working memory. Adaptive functioning level was not significantly predicted by EF scores.

**Conclusions:**

The present study highlighted domain-specific atypical trajectories of EF in individuals with 22q11.DS and explored the link with psychotic symptoms. However, no relation between EF and adaptive functioning was observed.

## Background

Executive functions (EF) can be described as interrelated high-level cognitive processes that play a leading role in formulating goals, planning how to achieve them, and carrying them out successfully [1, 2]. In the cognitive literature, there is evidence for the fractionation of EF [1, 3, 4]. Multiple EF domains are included under the EF umbrella (i.e., initiation of activity, cognitive flexibility, planning, self-regulation, or working memory), all of which play a chief role in day-to-day autonomy and are relevant to most aspects of life [3]. EF emerge early in childhood and continue to develop up to the beginning of adulthood, with each individual domain developing at a different pace [5], making EF a complex topic of study.

22q11.2 deletion syndrome (22q11.2DS) is a genetic disorder, one of the most common multiple anomaly syndromes in humans [6], and is reported to occur in approximately 1 in 4000 live births [7]. Nevertheless, recent studies suggest that its occurrence could be even higher [8]. The phenotype encompasses physical features like heart anomalies, cleft palate, or structural brain anomalies, as well as cognitive and behavioral features, including high rates of psychiatric disorders [6, 9]. A large proportion of affected individuals exhibit early onset psychosis [10], and 22q11.2DS is associated with increased risk for developing schizophrenia during adulthood [11]. This makes 22q11.2DS the best homogeneous human model for studying early risk factors and interventions for psychosis [12]. The cognitive profile in 22q11.2DS is characterized by intellectual functioning (measured by intelligence quotient (IQ)) in the borderline range (70–79), with noted deficits in numeracy, visuospatial processing, attention, and multiple executive function domains [12–15]. Variability in the cognitive profile can be observed between individuals, as well as within individuals over the years (Philip and Bassett [9]). For this reason, it appears necessary to study 22q11.2DS using a developmental approach.

Several studies have investigated EF in 22q11.2DS (e.g., [16–18]) in order to characterize the neurocognitive profile in this population. However, the measures used in these studies were generally part of larger batteries examining memory, intelligence, visuospatial processing, or language and were not specific to EF. Although they report EF as a relative weakness in the cognitive profile of individuals with 22q11.2DS, it is still unclear which domains are more or less affected and how each one develops over time. A few studies have examined a single component of EF to identify specific mechanisms leading to executive impairments. One EF domain, which has received a significant amount of attention in 22q11.2DS, is inhibition. McCabe et al. [19] examined pre-pulse inhibition in adolescents and found increased antisaccade errors and a trend toward impaired sensory motor gating, indicating a dysfunction of inhibition pathways in the syndrome. Likewise, Shapiro et al. [20] detailed the processes responsible for successful inhibition in children. The authors found that, when compared to controls, reactive inhibition (stopping) was impaired in individuals with 22q11.2DS, whereas proactive inhibition (anticipatory stopping) was preserved. Azuma et al. [21] focused on a different EF domain and observed significant spatial working memory deficits in children with 22q11.2DS. Together, these data highlight specific EF impairments in the syndrome but do not provide any information about the way EF domains develop in 22q11.2DS.

To our knowledge, only one study to date has assessed several types of EF within the same cross-sectional study of young individuals (7 to 14 years old) with 22q11.2DS [22]. The results of Shapiro et al. point to deficits in response inhibition, cognitive flexibility, and working memory (both verbal and non-verbal), even after controlling for the influence of intellectual functioning. In addition, the authors identified atypical development of both response inhibition and cognitive flexibility in children with 22q11.2DS compared to typically developing individuals. Altogether, this study suggests that EF impairments in the syndrome have a complex trajectory and are not simply a by-product of developmental delay. However, because of the cross-sectional nature of the study, the authors did not examine true developmental trajectories of the EF domains, an especially important step in 22q11.2DS research due the cognitive heterogeneity in the syndrome. Moreover, given the prolonged development of EF and their underlying prefrontal brain regions up to early adulthood [e.g., 23], it is interesting to investigate EF in an age range as broad as possible to understand the developmental context for each individual’s trajectory. To shed light on these lingering questions, we sought to delineate the developmental trajectories of several EF domains using longitudinal data acquired in a large cohort of individuals with 22q11.2DS aged 6 to 26 years. Previous studies have shown that differences in developmental trajectories between two groups can be described in different ways: (1) same general shape but the curve is shifted along the age axis, with the peak value attained at a later age; (2) difference in tempo with spurts at one or several time points; and (3) trajectory lacking shape [24].

Research on 22q11.2DS often focuses on the search of predictive aspects of development to stave off later outcomes. One of the main challenges is to identify, as early as possible, the factors that influence outcome as well as the emergence of psychotic symptoms in order to facilitate the development of specific interventions strategies. Previous studies have shown consistent EF alterations in patients with schizophrenia [25]. Associations between executive dysfunctions and symptoms of psychosis were also reported in this population, especially with negative symptoms [26, 27]. Specifically, significant associations were found between negative symptoms and inhibition [28]. One previous study in 22q11.2DS also found associations between negative symptoms and multitasking skills [29]. However, no longitudinal studies have been conducted on this topic in this population so far.

Initiating behavior at the right time, knowing when to stop oneself, organizing one day, or planning ahead to be more efficient in different activities are examples of how EF skills are crucial for adaptive behavior in the daily life [30]. Therefore, the third aim of this study was to describe the relationship between different EF domains and measures of adaptive functioning. Often considered as important outcome measures, IQ scores reflect acquired knowledge and test performance, whereas adaptive functioning is often overlooked [31]. Closely correlated with IQ, but with higher ecological validity, adaptive functioning measures, such as the Vineland Adaptive Behavior Scales (VABS) [32], provide information on daily life that can help to gauge a person’s autonomy. In contrast to what is observed in typically developing individuals, previous studies showed that IQ was not strongly correlated to adaptive functioning in 22q11.2DS and that adaptive functioning scores were usually lower than what is expected considering their intellectual level [31, 33]. This underscores the importance of examining the cognitive deficits that may alter adaptive functioning in this population.

For the present study, we proposed three main hypotheses: first, we hypothesized that individuals with 22q11.2DS would perform less well than the control group on all executive domains and that the developmental trajectory of the 22q11.2DS group would be different from the control group across all domains. Based on previous cross-sectional findings [22], we expected to find differences not only in terms of delay but also in the shapes of the trajectories (very little evolution with age or early decline). Second, we hypothesized that executive deficits would be involved in the emergence of symptoms of psychosis, especially negative symptoms, and that the developmental trajectories of the executive domains would differ between participants who will present with psychotic symptoms and those who will not. Third, since adaptive functioning depends on executive aspects of cognition [30], we hypothesized that scores in EF domains would predict adaptive functioning scores in individuals with 22q11.2DS.

## Method

### Participants

One hundred ninety-five participants aged 6–26 were recruited as part of a 22q11.2DS longitudinal study. Ninety-five of them were diagnosed with 22q11.2DS and 100 were typically developing controls, including siblings (56 %) and community controls. The two participant groups were commensurate for gender and age when compared at the first time points but differed on full scale IQ (Table 1). Participants were recruited using advertisements in patient association newsletters and word-of-mouth. The presence of a 22q11.2 deletion was confirmed using quantitative fluorescent polymerase chain reaction (QF-PCR). Written informed consent, based on protocols approved by the Institutional Review Board of the Department of Psychiatry of the University of Geneva Medical School (Switzerland), was obtained for all participants and their parents (if the participant was younger than 18 years old).

**Table 1:** Participant characteristics, psychiatric diagnosis and psychotropic medication

			Diagnostic group		Comparison			
			22q11.2DS	Controls	*t* test	Pearson’s chi-square	ANOVA	*p* value
*N*			95	100				
Gender (male (%))			45 (47.36 %)	48 (48 %)		0.008		0.930
Age at first time point (mean (SD))			12.80 (4.23)	13.17 (4.43)	0.596			0.552
Full-scale IQ at first time point (mean (SD))			70.71 (12.27)	110.37 (13.62)			454.57	<0.001
VABS outcome measure at last time point (mean (SD))		ABC score	66.73 (12.53)					
		Communication score	71.17 (17.00)					
		Daily living skills score	71.06 (15.22)					
		Socialization score	73.83 (14.51)					
Psychiatric diagnosis (*N* (%))		Simple phobia	42 (44.21 %)					
		Attention deficit disorder	36 (37.89 %)					
		Generalized anxiety	16 (16.84 %)					
		Major depressive episode	13 (13.68 %)					
		Psychosis	8 (8.42 %)					
		Obsessive-compulsive disorder	7 (7.37 %)					
Psychotropic medication	Total		39 (41.05 %)					
	Categories	Methylphenidate	26 (66.66 %)					
		Antidepressants	12 (30.77 %)					
		Antipsychotics	7 (17.95 %)					
		Antiepileptic	7 (17.95 %)					
		Anxiolytic	3 (7.69 %)					

In total, 352 testing time points were acquired, 188 (53.41 %) for 22q11.2DS patients. Longitudinal data (ranging from two to four time points per participant) was available for many participants (55.38 %) (Table 2). For participants with at least two time points, the mean interval between consecutive visits was 3.68 years (SD = 0.87). For individuals with only one time point (44.62 %), 66 (75.86 %) of them either did not have the opportunity to return for a second assessment or dropped out of the study. Twenty-one had additional time points available that were excluded due to missing data (18; 22.45 %) or to fit the age range of the study (4; 4.60 %).

**Table 2:** Longitudinal data available per time points

	Number of individuals having at least				
	1 time point	2 time points	3 time points	4 time points	Total
22q11.2DS	95	59	29	6	189
Controls	100	49	12	3	164
All	195	108	41	9	352

### Materials

#### Cognitive functioning

As part of an ongoing research protocol, participants completed the Wechsler Intelligence Scale for Children (WISC-III) [34] or the Wechsler Adult Intelligence Scale (WAIS-III) [35] to measure general intelligence and reasoning abilities at each time point. Neuropsychological testing included the Conner’s Continuous Performance Test (CPT) [36] to evaluate attention and impulsivity, the Stroop task [37] as a measure of inhibition, and the semantic verbal fluency, as a measure of verbal fluency. As we were especially interested in the influence of age on these EF constructs, raw scores were always used.

To assess EF, we selected different variables to disentangle the following executive domains: working memory, inhibition (cognitive and motor), and verbal fluency.

Working memory was assessed using the Wechsler *Digit Span* subtest, backward part. In this task, participants were asked to repeat backward a gradually increasing set of numbers. Two types of inhibition were investigated: motor and cognitive. In the CPT, participants were instructed to press a button every time a letter appeared on the screen, except for the letter X where participants had to withhold their answer. Several variables are computed based on the participants’ performance, and three of them are typically considered as reflecting inhibition processes [36]. The first one is the *commission error score*, which records every time individuals respond erroneously to a non-target. The second one is *hit reaction time score,* defined as mean response time (in milliseconds) for all correct responses. Fast reaction times combined with an unusually high percentage of commission errors can indicate impulsivity. The third score is the *perseveration score*, defined as a response that occurs less than 100 ms after a stimulus. Since perseveration errors can occur for different and often unidentifiable reasons (pre-emptive responding, random responding, or a slow response to the preceding stimulus), we only used the first two scores. To measure the cost of cognitive inhibition in time, we computed an *inhibition ratio* score by dividing the raw score from the Stroop condition (participants have to name the color of the ink even though the word spells a different color) by the raw score in the color naming condition (participants are instructed to name rectangles of colors as fast as possible). This score reflects the cognitive cost of inhibiting the reading process. A ratio value close to 1 indicates a lesser cost of inhibition. Finally, we assessed verbal fluency using the semantic verbal fluency test, animal category. In this task, participants were asked to name as many animals as possible in 1 min, without repetitions (e.g., lion, lioness) or proper nouns. This specific category was chosen to ensure that the task difficulty was similar between younger and older participants, since the animal category is not as dependent on reading and writing skills as letter categories. In this task, several variables can be extracted to reflect EF. The first one is the number of word produced, which reflects the capacity to actively search for an answer. The second one is repetitions and perseveration, which are used as an indicator of monitoring and mental flexibility. Variability in the distribution of repetition and perseveration scores was low and strongly deviated from a normal distribution. Therefore, we decided to consider only the number of word produced. This variable is not solely an executive measure and is influenced by lexical level. As a supplementary analysis, we examined the developmental trajectory of a “pure” measure of lexical level, namely the French version of the Peabody Picture Vocabulary Test (PPVT) [38].

#### Clinical assessment

All 22q11.2DS participants and their parents were interviewed separately by a trained psychiatrist using the computerized Diagnostic Interview for Children and Adolescents-Revised (DICA-R) [39] and the Structured Clinical Interview for DSM-IV Axis I (SCID-I) [40]. Psychiatric diagnoses and psychotropic medication taken during testing are included in Table 1. Participants who received the same medication at several time points were only counted once. Information about psychotropic medications was divided in five distinct categories (methylphenidate, antidepressants, antipsychotics, antiepileptic drugs, and anxiolytic medications).

Presence/absence of psychotic symptoms at any time of testing was assessed with the Positive and Negative Syndrome Scale (PANSS) [41]. Both positive and negative symptoms were examined individually using the a priori positive and negative dimensions of the PANSS. For each symptom dimension, the 22q11.2DS sample was split in two using the following cutoff: participants with at least one item scored 4 or higher (i.e., moderate to severe intensity) were classified as presenting positive/negative symptoms, whereas the remaining participants composed the group with no positive/negative symptoms.

#### Adaptive functioning

Parents of 89 individuals with 22q11.2DS (89.47 %) were interviewed using the Vineland Adaptive Behavior Scales (VABS) [32] to provide information about participants’ adaptive behavior. Data were missing for six individuals. For individuals with several time points, we used data from the first time point available. In addition to the adaptive behavior composite score (ABC), the VABS measures three domains of adaptive behavior: communication, daily life functioning, and socialization. Age appropriate standardized scores were used (*M* = 100; SD = 15). For the four individuals older than 18 years old, we used the norms from the upper age level, as suggested in the interview manual.

### Statistical analyses

To quantify developmental trajectories of EF domains in individuals with 22q11.2DS and typically developing controls, we examined between-group differences using mixed models regression analyses, as described in previous studies by our group [42, 43]. This technique allowed us to model the within-subject factor as a nested variable [44]. For each variable, different models (constant, linear, quadratic, or cubic) were fitted using the *nlmefit* function in MATLAB R2011b (MathWorks). We employed a Bayesian information criterion (BIC)-based model selection method, one of the most powerful model selection methods for mixed models [45]. Statistical significance for the differences in trajectories between groups was assessed using a likelihood ratio test. The outcome of these analyses allows us to either identify shape differences (i.e., curves that do not follow the same path) or intercept differences (i.e., curves that follow a parallel path but not on the same intercept) between the two groups.

To ensure that observed differences were not related to intellectual disability, we separated the 22q11.2DS sample in two groups according to full-scale IQ scores at the first time point (“lower than 70” (*N* = 46) vs. “higher than 70” (*N* = 49) groups). We subsequently conducted the same analyses comparing the “higher than 70” group to the controls and the “lower than 70” group to the “higher than 70” group.

In order to examine the relationship between psychotic symptoms and EF domains in the 22q11.2DS group, we compared EF trajectories of individuals who developed psychotic symptoms from those who did not. Both groups were compared using mixed models regression analyses. Positive and negative symptoms were examined separately.

Finally, we investigated the predictive value of EF by conducting hierarchical stepwise regression analyses using the VABS composite score or the domain scores as the dependent variable and the EF domain scores as independent variables. To avoid multicollinearity between EF domains, one EF score per domain was selected: Stroop inhibition ratio, digit span backward, and verbal fluency. Full-scale IQ was added in the model in the first step and selected EF scores in the second step. These analyses were performed using SPSS version 22.

## Results

### Longitudinal analyses

**Fig. 1:**
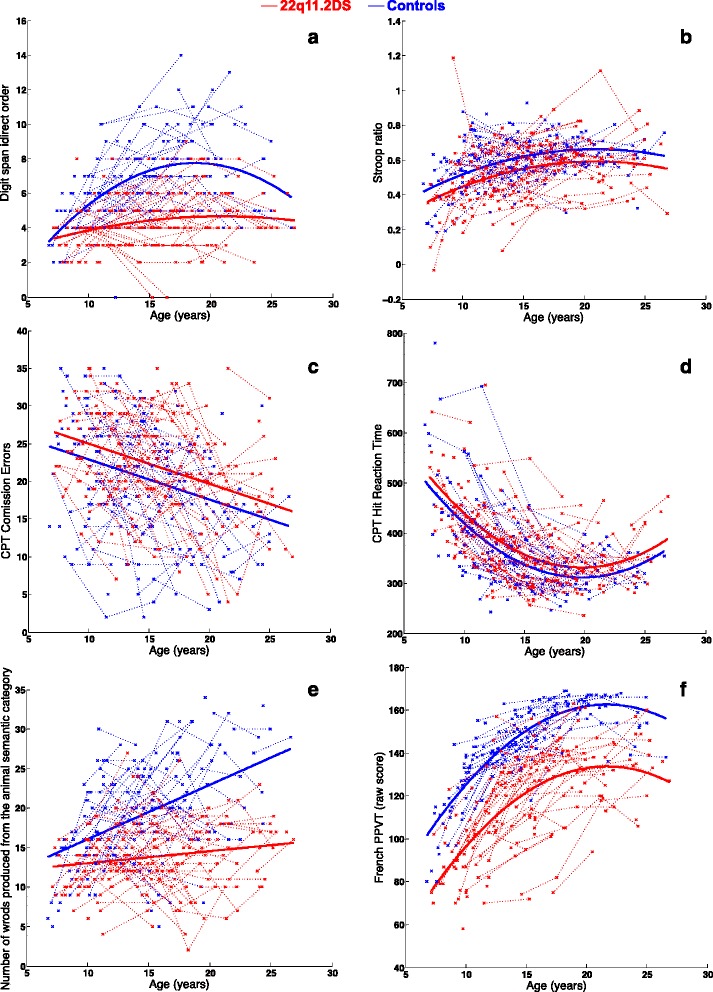
Developmental trajectories of **a** working memory (digit span indirect order), **b** cognitive inhibition (Stroop ratio), **c** motor inhibition (CPT Commission errors), **d** motor inhibition (CPT hit reaction time), **e** verbal fluency (animal fluency), and **f** vocabulary (French PPVT). The data points from a single subject are connected by a *dotted line*. The *solid lines* show the model fitted. Data from the 22q11.2DS group are displayed in *red* and controls are colored in *blue*

We compared the developmental trajectories of EF in individuals with 22q11.2DS and controls (Table 3). We observed significant differences in the shape of the groups’ trajectories with age for the working memory test (*p* < 0.004) and verbal fluency (*p* < 0.001). The control group demonstrated consistently higher scores. The intercepts were significantly different for the inhibition measures (*p* < 0.025). All tests survived a Benjamini-Hochberg [46] correction for multiple comparisons (*p* < 0.025). Most of the curves fitted a quadratic model of change with age (Fig. 1). For the 22q11.2DS groups as well as the control group, both working memory and cognitive inhibition (Stroop ratio) increased during childhood and peaked during early adulthood (18–22 years old), after which point we observed a gradual decrease. The CPT hit reaction time displayed the opposite pattern, with an initial decrease from childhood to early adulthood and then a subsequent increase. Verbal fluency and CPT commission errors fit linear increasing and linear decreasing models of change with age, respectively. Supplementary analyses comparing trajectories of vocabulary performance (French PPVT) with age exhibited no significant difference in terms of shape (*p* = 0.087) but a significant difference in terms of intercept (*p* < 0.001). These results exhibit a different pattern of development from the verbal fluency task.

**Table 3:** Differences in longitudinal trajectories between 22q11.2DS and controls

		Model fitted	Shape *p* value	Intercept *p* value
Working memory	Digit span indirect order	Quadratic	*<0.001*	n.a.
Inhibition	CPT commission errors	Linear	0.594	*0.025*
	CPT hit reaction time	Quadratic	0.387	*0.020*
	Stroop ratio	Quadratic	0.097	*<0.001*
Verbal fluency	Animals	Linear	*<0.001*	n.a.
Vocabulary	French Peabody Picture Vocabulary Test	Quadratic	0.087	*<0.001*

Significant values after correction for multiple comparisons with Benjamini-Hochberg procedure (i.e., where *p* < 0.025) are displayed in italics

*n.a.* not applicable

We then removed from the 22q11.2DS sample all individuals with a full scale IQ score lower than 70 (see “Statistical Analyses” section) and repeated the mixed models regression analyses on EF variables. After a Benjamini-Hochberg correction (*p* < 0.016), the results were comparable to those reported above (see Table 3), except for the CPT measures (commission errors and hit reaction time) which were not statistically different from the controls (Table 4). Finally, when compared to each other, the *lower than 70* group did not significantly differ from the *higher than 70* group, except on verbal fluency, for which the *higher than 70* group had a higher intercept (*p* = 0.001) in a constant model (Table 5).

**Table 4:** Differences in longitudinal trajectories between 22q11.2DS with full-scale IQ higher than 70 and controls

		Model fitted	Shape *p* value	Intercept *p* value
Working memory	Digit span indirect order	Quadratic	*0.012*	n.a.
Inhibition	CPT commission errors	Linear	0.607	0.104
	CPT hit reaction time	Quadratic	0.607	0.174
	Stroop ratio	Quadratic	0.023	*0.003*
Verbal fluency	Animals	Linear	*0.001*	n.a.

Significant values after correction for multiple comparison with Benjamini-Hochberg procedure (i.e., where *p* < 0.016) are displayed in italics

*n.a.* not applicable

**Table 5:** Summary from hierarchical multiple regression examining predictive aspects of adaptive functioning scores on executive functioning domains

	Dependent variables				
Steps	Independent variables	*R* ^2^	*R* ^2^ change	*F* change	*p*
	VABS ABC score				
Step 1	Full-scale IQ	0.220	0.220	24.529	*<0.001*
Step 2	Executive domains	0.224	0.004	0.136	0.939
	VABS communication score				
Step 1	Full-scale IQ	0.323	0.323	41.506	*<0.001*
Step 2	Executive domains	0.335	0.012	0.514	0.674
	VABS daily living skills score				
Step 1	Full-scale IQ	0.078	0.078	7.387	*0.008*
Step 2	Executive domains	0.960	0.018	0.554	0.647
	VABS socialization score				
Step 1	Full-scale IQ	0.750	0.750	7.083	*0.009*
Step 2	Executive domains	0.101	0.025	0.793	0.501

Significant values are displayed in italics

### Influence of executive dysfunction on psychotic symptoms

Participants presenting with negative symptoms at any time point showed significant shape differences in the trajectories of the CPT commission errors and digit span indirect order scores compared to participants without negative symptoms (*p* < 0.025 after the Benjamini-Hochberg correction, see Table 6). The remaining EF variables did not significantly differ between the two groups. On the opposite, participants presenting with positive symptoms at any time point did not differ from those not presenting positive symptoms on any EF measure (see Table 7).

**Table 6:** Differences in longitudinal trajectories between 22q11.2DS with negative symptoms and without

		Model fitted	Shape *p* value	Intercept *p* value
Working memory	Digit span indirect order	Linear	*0.038*	0.710
Inhibition	CPT commission errors	Linear	*0.007*	0.627
	CPT hit reaction time	Quadratic	0.617	0.128
	Stroop ratio	Linear	0.440	0.816
Verbal fluency	Animals	Constant	0.668	n.a.

Significant values after correction for multiple comparisons with Benjamini-Hochberg procedure (i.e., where *p* < 0.025) are displayed in italics

*n.a.* not applicable

**Table 7:** Differences in longitudinal trajectories between 22q11.2DS with positive symptoms and without

		Model fitted	Shape *p* value	Intercept *p* value
Working memory	Digit span indirect order	Linear	0.448	0.271
Inhibition	CPT commission errors	Linear	0.222	0.580
	CPT hit reaction time	Quadratic	0.792	0.417
	Stroop ratio	Quadratic	0.868	0.675
Verbal fluency	Animals	Constant	0.078	n.a.

*n.a.* not applicable

### Adaptive functioning

Hierarchical multiple regressions controlling for full-scale IQ were used to investigate the links between EF and adaptive functioning. EF did not significantly predict VABS scores (all *p* > 0.05) (see Table 5).

## Discussion

The main goals of the present study were to describe executive dysfunction in 22q11.2DS, to examine developmental patterns in the syndrome compared to controls as well as the influence of psychotic symptoms on these patterns, and to identify the predictive value of EF on adaptive functioning. To achieve these goals, we used multiple measures of EF to describe the development of working memory, inhibition, and verbal fluency in a longitudinal study of 22q11.2DS individuals and healthy controls ages 6 to 26.

### Atypical developmental trajectories of specific EF domains

Lower performance was observed on all EF variables for participants with 22q11.2DS compared to controls. In the 22q11.2DS group, atypical developmental trajectories were observed for working memory and verbal fluency, whereas the shape of the inhibition measures’ trajectories did not differ between the two populations. These EF impairments are commensurate with previous studies examining working memory and inhibition [20, 22]; however, to our knowledge, this is the first study reporting verbal fluency alterations in the syndrome.

#### Development of verbal fluency

In typically developing children, verbal fluency, measured by the number of words produced during a specific time lap, improves with age [47] until 13 to 15 years old [1, 48]. Similarly, in our control sample, we observed a gradual increase in performance on the verbal fluency task, though we did not observe a peak around mid-adolescence (13–15 years). One possible explanation for this difference could be that a group of older controls with very high scores influenced the trajectory of our control group. By contrast, improvement with age in the 22q11.2DS group was minimal, suggesting that as affected individuals get older, their strategies to successfully initiate and produce words from a semantic category do not progress as quickly as for controls. Interestingly, our sample groups performed similarly on the verbal fluency task during childhood (6–8 years old) before between-group differences became greater with age, a seemingly banal observation that deserves careful consideration given that non-executive aspects (verbal memory disorders or lowered psychomotor speed) can affect verbal fluency (e.g., [49]). To ensure that the results reported here are mostly due to executive dysfunction, and not due to a lower lexical level in participants with 22q11.2DS, we conducted a secondary analysis on vocabulary performances. We observed different patterns of development for the word fluency task and the vocabulary task. This indicates that even though the lexical level of the 22q11.2DS group is significantly lower than controls, the developmental path is similar between both groups (see Table 3 and Fig. 1). Trajectories for both groups displayed a gradual increase in raw scores until the age of 20, indicating that the lexical stock in the 22q11.2DS group increases at the same pace as in the control group. The results observed for the vocabulary task are in contrast with the developmental trajectories obtained for the verbal fluency task, which exhibited a significant difference in shape. As displayed in Fig. 1, there was only a minimal improvement with age in the 22q11.2DS group. This implies that even though their lexical stock increases with age, the number of words correctly produced during the verbal fluency test remains (approximately) identical. Altogether, this analysis suggests that the atypical trajectory observed for the verbal fluency task reflects, at least partially, an executive dysfunction even though it is not a pure executive measure. A qualitative analysis of the productions (i.e., clustering of words, switch between clusters) would be an informative addition to future studies [48, 50].

#### Development of working memory

Verbal working memory, measured by a number repetition task (backward digit span), is another EF domain explored longitudinally in the present study. Our participant groups differed in the shape of their development on verbal working memory measures, indicating that this domain develops atypically in 22q11.2DS compared to controls. However, similar to the verbal fluency results, while the younger children (6–8 years old) were not especially different from the controls, participants with 22q11.2DS tended to reach a developmental plateau much faster than controls. These results contrast with previous findings suggesting that working memory develops typically within the syndrome (i.e., weaker performance but same progression as in the control group) [22]. This difference may be related to two important methodological discrepancies with Shapiro et al.’s study. First, the limited age range in the previous study (7 to 14 years old) may have made it difficult to observe changes occurring later in life. This is in accordance with our result that younger children with 22q11.2DS performed similarly to their typically developing peers on working memory tasks. Without the inclusion of older adolescents and adults in our sample, we would not have observed a developmental plateau in working memory. Second, Shapiro et al. adopted a cross-sectional design, which may have prevented the detection of atypical developmental trajectories in the 22q11.2DS group.

#### Development of inhibition

The final EF domain investigated in the present study was inhibition, which was evaluated using measures of the cognitive cost of inhibition (Stroop ratio) and impulse control (CPT commission errors and hit reaction time). The performance of 22q11.2DS participants on the inhibition measures exhibited a shape resembling that of controls, despite the fact that the 22q11.2DS group’s scores were significantly lower than those of the controls (i.e., significant intercept difference). Specifically, the pattern emerging from our analyses depicted an increase in inhibition capacities with age in 22q11.2DS, echoing what is observed in the control group. These results are in contradiction with previous findings reporting atypical developmental of inhibition in 22q11.2DS [22]. However, the methodological differences between the two studies (age range, task differences, longitudinal design) may, once again, account for these discrepancies. The same group of authors published a previous study examining the development of inhibition using a task that differentiated between the processes underlying response inhibition (proactive, reactive) [20]. The authors reported significant differences between these processes suggesting that the mechanisms underlying inhibition might be affected unevenly in the syndrome. In light of these previous findings, it may be that our tasks tap different underlying constructs than the tasks used by Shapiro et al. Future studies examining the different components of inhibition longitudinally would help explain these discrepancies.

In summary, our first hypothesis was only partially supported. 22q11.2DS individuals were impaired on all three EF domains compared to controls but exhibited atypical development on only two of those domains (working memory and verbal fluency).

#### Role of intellectual disability on EF measures

Post hoc analyses allowed us to disentangle the influence of intellectual disability on EF tasks in the present study. Even when individuals meeting the criterion for intellectual disability (full-scale IQ lower than 70 points) were removed from the 22q11.2DS sample, the trajectories of working memory, verbal fluency, and cognitive inhibition remained unchanged. This indicates that the different developmental trajectories (differences in shape) of working memory and verbal fluency between 22q11.2DS and controls are not only a by-product of intellectual disability. Furthermore, the intercept difference for the cognitive inhibition measure indicates a specific deficit rather than a consequence of intellectual disability. On the other hand, the developmental trajectory of motor inhibition (CPT commission errors and hit reaction time) no longer differed between the two groups, after the exclusion of individuals with intellectual disability. This lack of difference indicates that individuals affected by 22q11.2DS with an IQ higher than 70 have comparable motor inhibition than controls and that the subgroup with an IQ below 70 was probably driving the observation of poor impulse control.

Interestingly, when compared against each other, the 22q11.2DS subgroups did not significantly differ on EF measures, except for verbal fluency. A possible explanation for this result is that, as already mentioned before, verbal fluency is greatly influenced by non-executive functions that are also measured in IQ scales (e.g., vocabulary). Nevertheless, the fact that the *higher than 70* subgroup performed differently than controls indicates that verbal fluency is impaired in 22q11.2DS.

#### Relationship between executive dysfunctions and psychotic symptomatology

By comparing trajectories of individuals who displayed psychotic symptoms at any time point to those who did not, we found a link between certain executive domains and negative symptoms. Specifically, both for inhibition and working memory, performance of individuals with or without psychotic symptoms were very similar in childhood. However, improvement of these two processes with age was minimal for individuals with negative symptoms, whereas the group without symptoms improved significantly and regularly. These results seem to indicate that EF dysfunction exists prior to the onset of negative symptoms. On the opposite, no association was found with positive symptoms.

Hereby, we replicated that EF dysfunctions are specifically associated with the emergence of negative symptoms, whereas they are independent of positive symptoms in patients with schizophrenia [26, 28]. Also, these results are in line with a previous study by our group in 22q11.2DS, showing that negative symptoms were associated with deficits in multitasking skills [29]. In the present study, specific associations were found with the inhibition and working memory domains, which are involved in maintaining goals in memory and purposely implementing them at the right moment (e.g., in resisting dominant action scheme). It suggests that these processes could underlie the development of negative symptoms and is in accordance with previous conceptualizations of negative symptoms as a “pathology” of goal-directed behavior [51]. However, this hypothesis should be further examined, and these results need to be interpreted with caution since only a few aspects of EF were examined in the present study. In fact, positive symptoms could be influenced by an atypical development of other executive domains not considered in the scope of this article.

### EF and adaptive functioning

Contrary to our second hypothesis, we found no relationship between EF measures and adaptive functioning scores. It is possible that the absence of significant relationship is at least partially explained by our choice of EF tasks. Indeed, difficulties experienced in a test situation are not directly related to difficulties observed in the real world, such as those assessed in the VABS inventory [52]. Furthermore, examining only one process at the time, in a controlled experimental setting, free from distraction, may not be representative of day-to-day tasks that require the simultaneous use of several EF domains. For this reason, questionnaires targeting behavioral aspects of EF in a naturalistic context (i.e., Behavior Rating Inventory of Executive Functions (BRIEF) [53]) are usually poorly related to cognitive measures of EF in different clinical populations (i.e., [54]). In the field of 22q11.2DS, our group previously showed that poor multitasking abilities, as measured during a naturalistic experimental paradigm, were significantly associated with the VABS daily living skills domain [29]. Indeed, failure to multitask effectively may be a bigger hindrance to functional impairment than intellectual disability. These results indicate that to fully understand EF deficits in 22q11.2DS and to develop targeted interventions, it is necessary to use multiple measures with ecological validity to target core aspects of EF (i.e., inhibition, updating, cognitive flexibility).

### Limits, future directions, and clinical implications

Our work is not without critical limitations. First of all, the EF tasks used in the present study were selected retrospectively from a large longitudinal dataset. The chosen EF tasks involve other aspects of cognition and are not “pure” measures of EF. For example, working memory was only evaluated on its verbal component, whereas the visuospatial component is also very important. Furthermore, data on significant aspects of EF, such as cognitive flexibility or planning skills, were not available longitudinally, despite the fact that they are reported as weaknesses in this syndrome [16, 22]. Given that two out of the three investigated domains showed atypical development, other domains could be affected too. Future research should focus on collecting longitudinal data on a larger sample of tasks that specifically target and isolate EF domains. Furthermore, it would be important to integrate measures or questionnaires with ecological validity to truly capture the executive profile of this specific population. Finally, as illustrated in the current study, a great variability between individuals with 22q11.2DS was observed on the executive tasks. This heterogeneity begs the question of how to identify and characterize subgroups within the 22q11.2DS population. Future research should investigate this aspect in order to create more specific interventions.

Clinical implications of the results presented here are various. First of all, the data reported in this paper suggest that young children with 22q11.2DS (6–8 years old) have comparable performance to controls in some executive domains, but the gap between both groups widens progressively during adolescence. Furthermore, different executive domains do not display similar developmental patterns. Therefore, regular comprehensive neuropsychological assessments of EF should be conducted with individuals affected by 22q11.2DS to identify specific impairments. Secondly, if executive dysfunction is highlighted, specific interventions as well as environmental improvements could be implemented (e.g., planning and organization flowcharts, minimizing environmental interferences, break down information in small chunks). Finally, it remains to be examined whether cognitive remediation programs performed during childhood and focusing on EF have a beneficial impact on the development of EF later in life.

## Conclusions

In conclusion, we investigated the developmental trajectories of three executive domains in a large longitudinal cohort of individuals affected by 22q11.2DS and controls aged 6 to 26 years. We identified significantly lower performance on all three executive domains and atypical development of verbal working memory and verbal fluency in 22q11.2DS. Deficits in specific domains were related to future development of negative symptoms, but not positive. We further tested the predictive value of EF domains on adaptive functioning but observed no significant association.

## Abbreviations

22q11.2DS: 22q11.2 deletion syndrome
BIC: Bayesian information criterion
CPT: Continuous Performance Test
DICA-R: Diagnostic Interview for Children and Adolescents-Revised
EF: executive functions
IQ: intelligence quotient
PANSS: Positive and Negative Syndrome Scale
QF-PCR: quantitative fluorescent polymerase chain reaction
SCID-I: Structured Clinical Interview for DSM-IV Axis I
VABS: Vineland Adaptive Behavior Scales
WAIS-III: Wechsler Adult Intelligence Scale, third version
WISC-III: Wechsler Intelligence Scale for Children, third version
